# The need to redefine genomic data sharing: A focus on data accessibility

**DOI:** 10.1016/j.atg.2014.09.013

**Published:** 2014-09-28

**Authors:** Tempest A. van Schaik, Nadezda V. Kovalevskaya, Elena Protopapas, Hamza Wahid, Fiona G.G. Nielsen

**Affiliations:** DNAdigest, Future Business Centre, Kings Hedges Road, Cambridge CB4 2HY, United Kingdom

**Keywords:** Genomics, Data sharing, Collaborations, Data privacy, Data access

## Abstract

DNAdigest's mission is to investigate and address the issues hindering efficient and ethical genomic data sharing in the human genomics research community. We conducted contextual interviews with human genomics researchers in clinical, academic or industrial R&D settings about their experience with accessing and sharing human genomic data. The qualitative interviews were followed by an online survey which provided quantitative support for our findings. Here we present the generalised workflow for accessing human genomic data through both public and restricted-access repositories and discuss reported points of frustration and their possible improvements. We discuss how data discoverability and accessibility are lacking in current mechanisms and how these are the prerequisites for adoption of best practices in the research community. We summarise current initiatives related to genomic data discovery and present a new data discovery platform available at http://nucleobase.co.uk.

## 1 Introduction

Consistent decreases in the cost of DNA sequencing due to the success of next generation sequencing (NGS) technologies open up new horizons in clinical research and practice. Personalised medicine is expected to deliver faster diagnostics and provide more efficient treatments for a plethora of diseases (Karczewski, 2013). Despite the huge success of the Human Genome Project (HGP; U.S. Department of Energy Human Genome Project, 2014) there are multiple barriers that separate society from the potential benefits of the genetics clinic of the future. Human genomics research relies on the availability of genomic datasets that must be carefully selected, curated and analysed to test a hypothesis. Unfortunately, although a large amount of genomic data is generated around the world, individual researchers are still lacking access to the necessary amounts of the specific data they need to power their studies. Exemplary collaborative practices demonstrated during the realisation of the HGP do not reflect the state of data sharing in the community today: data sharing is not the default, but the exception. In general, many researchers and organisations have expressed support for increased data sharing but there are numerous well-known hurdles including technical, cultural, and legal restrictions limiting the extent to which data is shared (Hayden, 2013, Knoppers et al., 2014, Lim, 2014, Pickard and Swan, 2014).

Data sharing has continually been recognised as important, not only for the advancement of scientific knowledge, but also for the preservation of information: safeguarding against misconduct and verification of conclusions (Maurer, 2006, Tenopir et al., 2011). The initiatives promoting and facilitating data sharing throughout research communities are continuously growing, slowly changing the existing paradigms of scientific practices (Wellcome Trust, 2014a, Wellcome Trust, 2014b). A number of scientific journals already explicitly request raw data deposition to open repositories prior to publication and the NIH has recently issued a new data sharing policy that requires all publicly funded research output to be shared as quickly as possible (National Institutes of Health (NIH), 2014).

Data sharing in human genomics is a multifaceted challenge (Kosseim et al., 2014). Ethical considerations for use combined with the uniqueness of the genome of an individual require special precautions to enable sharing whilst protecting data privacy. The genome itself is personally identifiable information and standard anonymisation techniques may be insufficient, since even after anonymisation the remaining information may still be subject to re-identification of the individual (Erlich and Narayanan, 2014). At the same time, in studies involving human subjects, consent from data donors must be obtained to use their data for research (Beauchamp, 2011). The consent forms are still not widely standardised and may be either broad or narrow, which may open or restrict the potential usage of the data, respectively. Notably, the process of obtaining consent for research use may generate an implicit expectation that the collected data is widely shared and reused.

Here, we investigate the current extent of human genomic data sharing by examining the data handling processes and needs of human genomics researchers in different settings. We explore how researchers are including data access and data sharing in their current workflows and whether any bottlenecks need to be addressed to enable more efficient data collaborations.

## 2 Methods

Data on the current practices and experiences of accessing and sharing data for human genomics research was collected using a combination of qualitative and quantitative methods. The qualitative research was based on contextual inquiries in the form of guided interviews (Whiteside et al., 1988). A follow-up survey was used to provide quantitative sizing information (vide infra). It was sent directly to ~ 200 known human genetics researchers. Our contacts in this pool forwarded the survey to the Sanger and EBI internal mailing lists. In addition, we promoted the survey through our social media and sent it directly to the communications/office assistant of 158 research institutions and clinics working with genomic data of which ~ 75 were in the UK and the others from the rest of the world.

**Fig. 1:**
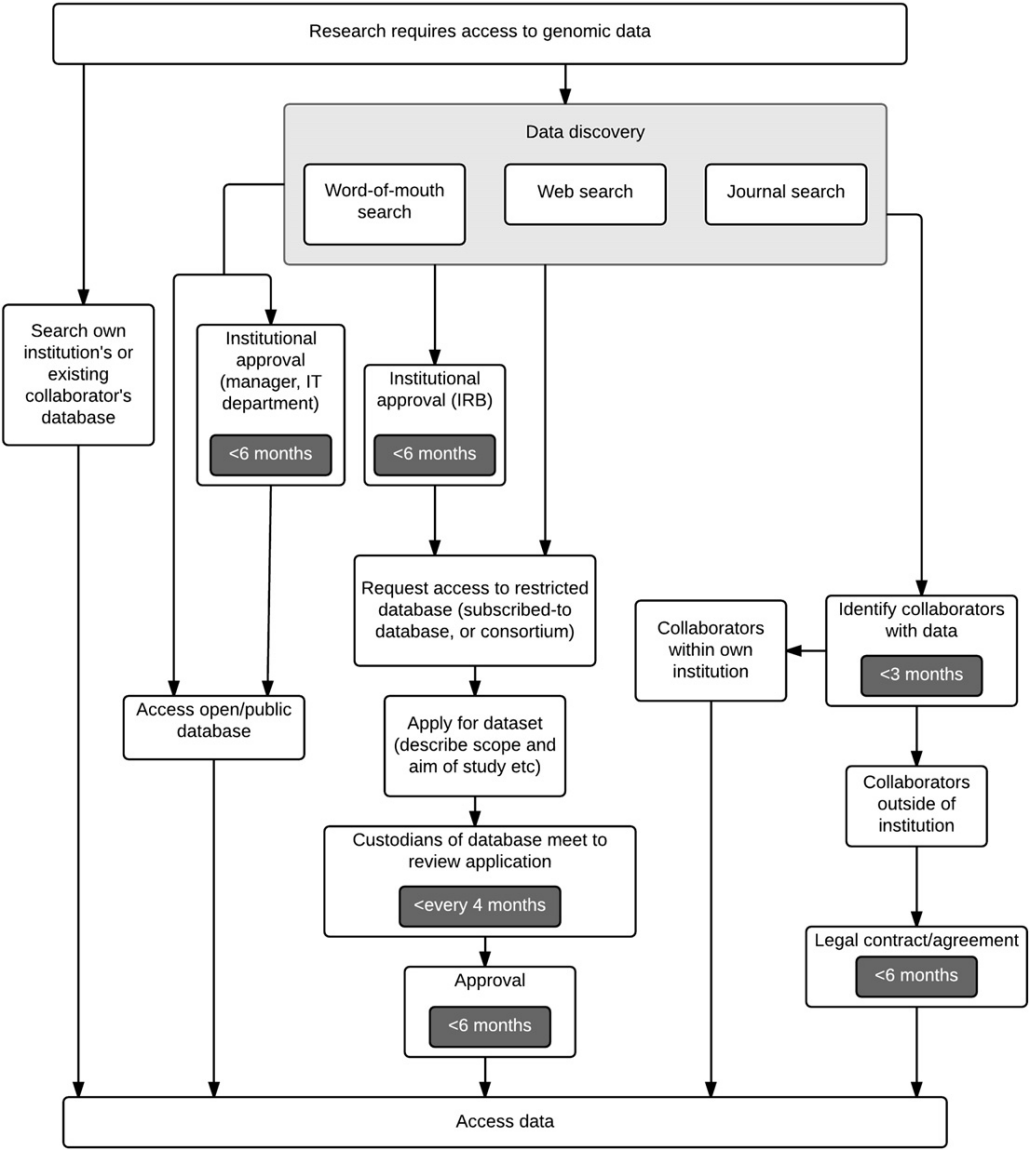
The generalised workflow that researchers follow to access human genomic data based on qualitative interviews conducted with 20 researchers in academia, or a clinical or industrial R&D setting. The main steps of the workflow are included and where possible, the duration of the steps according to the interview responses is indicated.

Participants for in-depth interviews were identified and contacted from the genetic researcher community in the Cambridge and London area within the UK, and in the Netherlands; where possible, the interviews were conducted in the researcher's own workspace. Interviews were conducted one-to-one with a total of 20 researchers using human genomic data either in a clinical, academic or industrial R&D setting. In a guided discussion lasting approximately 30–60 min, the researchers were questioned regarding their experience of i) finding and gaining access to data, ii) managing and storing data, and iii) sharing their own data. In addition, each interviewee was asked to draw a workflow diagram, indicating the steps involved in accessing and sharing data and the time required for each step. The interview responses and workflows were documented per respondent. By comparison of the workflow diagrams, common elements were identified and the generalised workflow for data access was generated (Fig. 1).

A survey on the same topic as the in-depth interviews was designed to collect quantitative information from a wider audience. The questions in the survey were designed as multiple-choice questions based on the answers collected from the first 10 interviews. We designed the survey to be conducted online^1^ with 17 mandatory questions related to researcher practices and experiences with data access and data sharing, five mandatory questions to collect demographics data and three optional questions regarding opt-in for re-contact regarding the outcome of the research. To increase the survey reach, we emailed the survey link to mailing lists in the genomics research community. By the time of submission of this paper a total of 652 individuals had visited the survey page and a total of 65 had completed the survey. Approximately half of the respondents were from the UK.^2^

**Fig. 2:**
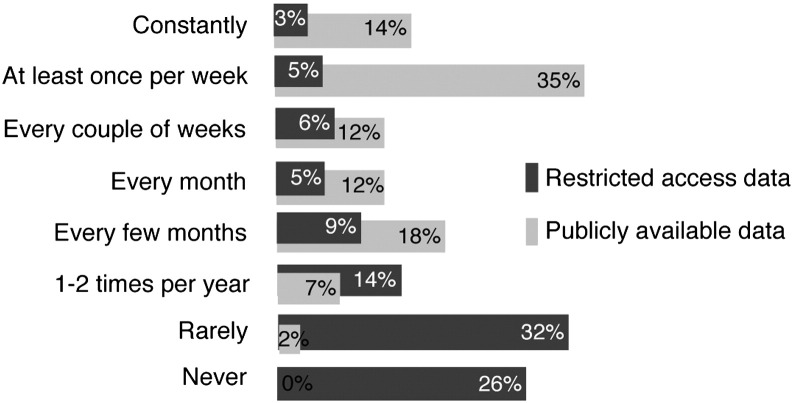
Survey responses to the question: “How often do you access datasets from public/restricted repositories?”. A total of 65 respondents completed the survey. The distribution of frequency of access was similar for all respondent groups independent of the affiliation (comparison between groups not shown).

The survey results were summarised per question into percentages, and for the questions related to frequency of data access we compared the frequency of public *versus* restricted-access data sets for industry *versus* academic researchers (Fig. 2). In the following sections, all percentages quoted are from the survey results. The results were plotted as bar charts using Microsoft Excel. The final results of the survey are available at: http://dnadigest.org/surveys/.

## 3 Results

### 3.1 Data access and data management

The interview results revealed many commonalities in the way genomic data is accessed and managed. These patterns point to a generalised workflow that researchers follow when searching for and accessing human genomic data. Institutions differ in which databases they access and how efficient their data access process is, but the common steps that all the interviewees follow in their practice are represented in Fig. 1. In most cases, there is a substantial lead time for accessing genomic data and the only two routes to fast data access involve using i) in-house databases or those of collaborators and ii) open/public access databases where no additional institutional approval is necessary. In the cases when a relevant dataset is neither present in an open database nor provided by collaborators, researchers will try to access data from a restricted-access database, such as EGA or dbGaP. This process of accessing data from a restricted-access repository was reported as a time-consuming and frustrating experience.

The following four bottlenecks were identified from the interview responses:

i) *Finding relevant and usable data (data discovery).* Searching for relevant data is haphazard for most interviewees and usually involves a general web keyword search, visiting one or more of their favourite databases, searching through a journal database and/or tracing the data referenced in a published article, or a word-of-mouth search which involves enquiring about data from colleagues and collaborators. One researcher said: “*About half my time is spent assessing data and talking to collaborators to see if it is relevant.*”
ii) *Getting authorisation to access data*. Once a dataset has been identified, some researchers need to make a case to their supervisor and/or IT department for the importance of the dataset and this request is usually escalated through the organisation. In some cases, even if approval is granted it may be for very limited use of the data under strict conditions. Additionally, if the data is being shared between institutions (e.g. academia and industry) it may take significant time to draw up a legal contract about intellectual property, ownership of results, publication authorship, responsibility for data, etc. As a result, a legal contract between collaborators and ethical approval can take up to six months: “*Inter-institutional collaboration can take many months because of the legal requirements of sharing data, in which time the research can change direction or the collaboration may be cancelled entirely.*”
iii) *Formatting data.* This step includes converting file types, standardising, performing quality control for reliability and relevance and customising data for specific applications. This is another time consuming process that can take up to one month: “*When I start a new research project, one of the biggest bottlenecks is searching for and formatting large amounts of data from multiple websites into a usable form because it is not standardised.*”
iv) *Storing and moving data*. Storing and moving data are often a problem because of the large size of genomic data sets and therefore require special arrangements, for example, transportation on a hard-drive: “*The size of genetic data (terabytes-petabytes) that needs to be downloaded, processed and moved is a burden.*”

In smaller organisations, genomics researchers are responsible for handling data themselves and therefore need to spend a significant amount of their time completing steps i)–iv) for data access, whilst larger organisations usually have a supporting team of bioinformaticians or technicians that may assist steps ii)–iv). All interviewees reported that the resources and time required to access data are a hurdle. In some cases, delays in accessing data may be so long and/or the process so complicated that the research question can become outdated before the data is actually retrieved or used. In academic research, data sharing may happen with collaborating groups, either with formal guidelines as a part of research consortia or informally between researchers working on a project together. Researchers in industry mentioned that any collaboration that includes sharing of results or data with external groups requires legal agreements to determine the rights to any intellectual property resulting from the collaboration. This requirement was indicated as a barrier for sharing due to the resources and time involved to put a legal agreement in place.

Several researchers expressed that the time and effort involved in accessing certain types of data have been built into their workflow: they know that projects involving certain kinds of data cannot be time critical, they always work in parallel, jobs have been created specifically to handle data, and long periods of waiting are part of the *status quo*. In some cases, instead of initiating the lengthy process of finding and accessing data, the problems and delays are avoided by simply limiting research questions to those that can be answered with existing datasets.

All interviewees mentioned that usefulness of genomic data for their project is determined by quality of both data and metadata and how well this metadata is curated and organised. The metadata may include information about the research subject (a healthy individual or a patient), type of disease and ethnic group, and technical information on the way the sample was obtained and handled to generate the data.

The majority of the interviewees communicated that having access to more data (possibly even from repositories beyond their knowledge) would stimulate their research. Some clinical researchers reported frustration at having to search for clinical data that they felt had probably been found and accessed by colleagues in their institution before, but not made available for sharing within the institution. Almost all interviewees stated that tools that would allow discovery of new datasets and facilitate access to such datasets would open up more research possibilities and also, importantly, improve the statistical power and significance of their results.

### 3.2 Data sharing

Interviewees generally had limited experience of making their own data available. The process of sharing data through deposition in restricted-access repositories was reported to be difficult and time-consuming mainly because data has to be formatted to meet strict and sometimes inflexible requirements. In addition, completing the forms describing the scope of the consent for data usage and obtaining the required institutional approvals may be a complicated and resource-intensive process. Regardless of existing data sharing policies which vary across institutions, even when researchers are authorised to share data they report reluctance to do so because of the amount of effort required. They did unanimously agree that the research community would benefit from more data sharing. In general, they only share data when forced to through publication, when specifically requested to do so by another researcher, or when officially collaborating in order to share data. One interviewee responded: “*My perception is that sharing human genomic data would be difficult but I would be willing to go to greater lengths to share data because I believe it is important; more data sharing would open up new research opportunities and promote transparency and reproducibility in results*”.

There was a clear difference between the three categories of researchers regarding their reasons for not making more of their own data available to others even when they have the authority and consent to do so. Clinical geneticists cited a lack of time because their main priority is diagnosing patients. Industrial researchers cited a lack of time because of the pressure to meet the deadlines in their job. Researchers in academia cited both a concern about the potential loss of future publications once unpublished data is shared, and the lack of time and incentive to share data as this does not contribute to their publication record. Researchers from all categories felt that they lacked sufficient resources to make their data available.

### 3.3 Survey responses

The results of the online survey, containing questions addressing the issues we identified through the interviews, showed very good agreement with the findings described above.

#### 3.3.1 The value of data sharing

The majority of the survey respondents indicated that they perceive a lot of value from sharing data and making their data available: “Access to more data means more statistical power for validation” (89% agree), “Access to more data means better representation of genetic variation” (83% agree) and “Sharing data reduces duplication of effort” (83% agree).

#### 3.3.2 Making data available

Although the respondents demonstrated broad agreement with the advantages of data sharing, 40% indicated that they share their data only internally or with collaborators on the same project and 31% indicated that they share their data only after a manuscript has been submitted for publication.

#### 3.3.3 Accessing existing datasets

The frequency of accessing different data repositories varied greatly between publicly available and restricted-access databases. 49% of the respondents indicated that they access publicly available datasets at least once per week, in stark contrast to restricted-access data sets which only 8% of respondents would access at least once per week, 46% would access twice per year or less, and 26% said they would never access restricted-access data sets (Fig. 2). The top-five most accessed resources for human genomic data are 1000 Genomes (indicated by 63% of survey respondents), UCSC (61%), ClinVar (33%), GEO (30%), and COSMIC (26%). Notably, all these resources are free and open access databases. The most-accessed restricted-access repository was dbGaP (24%). Information about other databases is available at the survey results page (http://dnadigest.org/surveys).

#### 3.3.4 Data access bottlenecks

When asked about the bottlenecks in accessing data, 57% mentioned compatibility issues, 52% mentioned the approval process from the repository holding the data, and another 52% mentioned the size and the time required to download data. Nearly half of the survey respondents indicated that data discovery on its own consumed at least 20% of their time in relation to a research project.

#### 3.3.5 Respondent demographics

46% of the survey respondents were from the UK, 20% from the USA and 34% from the rest of the world. Approximately 57% of them indicated an academic affiliation. The research areas of the respondents were mainly complex diseases (34%), rare diseases (29%), cancer (26%) and diagnostics (25%); several respondents indicated multiple areas of research.

## 4 Discussion

The results of our interviews and survey demonstrate that there is a strong desire amongst researchers to access genomic data beyond what is available at their institution. In addition, our respondents expressed a willingness to make data available for sharing, provided that they have consent and permission to do so. However, they mentioned a number of difficulties regarding finding, accessing and using available data resources. The respondents reported that the effort and resources required for both accessing data and for making data available through current mechanisms are limiting their adoption of data sharing practices. From our survey results we noted a stark contrast between the usage of public versus restricted-access datasets which we suggest is a direct result of the effort required to access these datasets. The voiced concerns regarding the accessibility and general usability of restricted-access repositories echo recent findings in a survey of the dbGaP user base (Simpson et al., 2014). We see the difficulty of both data access and data sharing as an indicator of a general problem with current data sharing mechanisms: we believe that the ease of use, discoverability, availability and accessibility of data resources are crucial for promoting and facilitating data sharing for the genomics research community.

The challenges of data sharing have been discussed extensively in the human genomics research community, since sharing of samples, data and results is an essential building block for progressing the body of knowledge in the field (Callier et al., 2014). Several organisations including the Research Data Alliance (RDA; https://rd-alliance.org) and the Human Variome Project (HVP; http://www.humanvariomeproject.org) have made great efforts to promote capturing and sharing of research data, but hurdles in sharing genomic data still remain. In 2013, the Global Alliance for Genomics and Health (GA4GH; http://genomicsandhealth.org) was established and has since engaged more than 180 institutions and organisations in working groups to address the challenges of regulatory restrictions, ethics, clinical demands, data representation, storage, analysis, and security related to genomic data sharing.

DNAdigest (http://dnadigest.org) is a charity that takes part in this effort by organising collaborative workshops with the research community to develop new open source tools and prototype solutions. The first output of these efforts is a data discovery platform which is now under development by the social enterprise Nucleobase. The objective of this platform is to facilitate the data discovery process by making it a social and collaborative effort, which benefits not only an individual researcher but also their peers and collaborators. The first beta-version of this platform is available at http://nucleobase.co.uk.

Other initiatives that are implementing various approaches to improve the discoverability of genomic data across locations include: the Beacon project and the Matchmaker exchange (from the Global Alliance for Genomics and Health working groups), the Leiden Open Variation Database (LOVD) (Fokkema et al., 2011), ClinVar (Landrum et al., 2014), Cafe Variome (http://cafevariome.org) and PhenomeCentral (https://phenomecentral.org). Some of these initiatives focus on identification of specific genetic variants within public or restricted-access datasets (the Beacon project and Cafe Variome), others focus on reported correlations between variants and disease (LOVD and ClinVar), and others focus primarily on matching of phenotype information for individual samples to initiate contact between clinicians (Matchmaker exchange and PhenomeCentral). The DNAdigest data discovery platform is a portal to discover the existence of datasets across public and restricted-access repositories based on their metadata, descriptions and annotations contributed by the community. This approach offers complementary functionality to the aforementioned initiatives.

## 5 Conclusion

We identified the current challenges and bottlenecks in genomic data sharing and data access through in-depth qualitative interviews combined with an online survey designed to quantify the extent of genomic researchers' experience with data access and data sharing. We found that the steps involved in both data access and data sharing through restricted-access repositories are perceived as time-consuming and difficult. We conclude that availability, discoverability and accessibility of data resources are a critical first step to improve data sharing in genomics.

We think that it is important to continuously assess available solutions which facilitate data sharing/access and promote the mechanisms and practices that make the greatest impact. We believe that there is a need for more activities to develop and promote best practices before genomic data sharing will become the default rather than the exception.

## Footnotes

1 The survey was designed using the online survey tool Typeform (http://www.typeform.com).

2 The final results from the survey with complete demographics are available at: http://dnadigest.org/surveys.
